# Prestimulus EEG Oscillations and Pink Noise Affect Go/No-Go ERPs

**DOI:** 10.3390/s25061733

**Published:** 2025-03-11

**Authors:** Robert J. Barry, Frances M. De Blasio, Alexander T. Duda, Beckett S. Munford

**Affiliations:** Brain & Behaviour Research Institute, School of Psychology, University of Wollongong, Wollongong 2522, Australia; francesd@uow.edu.au (F.M.D.); aduda@uow.edu.au (A.T.D.); rm798@uowmail.edu.au (B.S.M.)

**Keywords:** pink noise, white noise, *PaWNextra* algorithm, oscillations after removal of noise, prestimulus oscillation components, poststimulus ERP components, equiprobable Go/No-Go task, behaviour, temporal and frequency PCA, brain dynamics

## Abstract

This study builds on the early brain dynamics work of Erol Başar, focusing on the human electroencephalogram (EEG) in relation to the generation of event-related potentials (ERPs) and behaviour. Scalp EEG contains not only oscillations but non-wave noise elements that may not relate to functional brain activity. These require identification and removal before the true impacts of brain oscillations can be assessed. We examined EEG/ERP/behaviour linkages in young adults during an auditory equiprobable Go/No-Go task. Forty-seven university students participated while continuous EEG was recorded. Using the *PaWNextra* algorithm, valid estimates of pink noise (PN) and white noise (WN) were obtained from each participant’s prestimulus EEG spectra; within-participant subtraction revealed noise-free oscillation spectra. Frequency principal component analysis (f-PCA) was used to obtain noise-free frequency oscillation components. Go and No=Go ERPs were obtained from the poststimulus EEG, and separate temporal (t)-PCAs obtained their components. Exploratory multiple regression found that alpha and beta prestimulus oscillations predicted Go N2c, P3b, and SW1 ERP components related to the imperative Go response, while PN impacted No-Go N1b and N1c, facilitating early processing and identification of the No-Go stimulus. There were no direct effects of prestimulus EEG measures on behaviour, but the EEG-affected Go N2c and P3b ERPs impacted Go performance measures. These outcomes, derived via our mix of novel methodologies, encourage further research into natural frequency components in the noise-free oscillations immediately prestimulus, and how these affect task ERP components and behaviour.

## 1. Introduction

### 1.1. Background

Electroencephalography (EEG) records limited information about our brain activity from the scalp’s surface, often discussed as a mix of waves or oscillations. Başar [1] described these as “the rhythmic and/or repetitive electrical activity generated spontaneously and in response to stimuli by neural tissue in the central nervous system” (p. 291). The frequency distribution of the EEG—usually obtained via Fourier analysis—has been divided into various bands (delta, theta, alpha, beta, and gamma) that have long been explored in relation to consciousness and performance in different populations and clinical states.

Cognitive neuroscience has shown increasing interest in linking EEG oscillations to aspects of perception and cognition in both neurotypical and clinical populations. In this context, our lab has concentrated its efforts on a simple two-choice reaction time (RT) task, the equiprobable auditory Go/No-Go task, which can be performed well by a wide range of participants, from young children to older adults. We initially developed a processing schema with neurotypical young adults [2] to link event-related potentials (ERPs) and behaviour in this task. We decomposed our ERPs using temporal principal component analysis (t-PCA) following suggestions derived from Donchin [3]. Our research has repeatedly indicated that the early P1 and N1 components marking the initial stimulus evaluation processing stages are similar in both Go and No-Go ERPs. The subsequent P2/N2 components mark identification of the stimuli as Go or No-Go, and the processing chains then diverge. The Go chain is marked by the P3b correlate of the active response, and the No-Go chain by the P3a as processing winds down without active responding. Subsequent slow wave (SW) components appear to reflect evaluative processing of that decision. We have confirmed and extended this schema in young and older adults [4], and in children [5,6]. It has also been explored in relation to development between children and adults [7], and in younger and older children [8]. Over the years, these studies have been accompanied by stepwise improvements in EEG/ERP recording/processing techniques, and optimisations in t-PCA and statistical analyses.

Early brain dynamics studies used traditional band limits to define EEG amplitudes immediately prestimulus and explored their effects on peak-picked ERPs in various paradigms. Prestimulus delta and/or theta activity was linked with amplitudes of N1–P2 [9,10], and P2 and N2 [11]; while prestimulus alpha was associated with N1 [12], N1–P2 [9,13], N2–P3 [13], and P3 [14] amplitudes. We continued this line of research, particularly focussed on the dominant N1 and P3 components of the equiprobable auditory Go/No-Go paradigm; this is the paradigm utilised in the present study. We have reported an inverse effect of delta (1–3 Hz) on Go and No-Go N1, and a direct effect on Go and No-Go P3, while theta (4–7 Hz) had direct effects on Go N1 and No-Go P3 and inversely modulated No-Go N1 and Go P3 [15]. Prestimulus alpha (8–13 Hz) showed no effects on N1 but direct effects on both Go and No-Go P3; conversely, beta (14–24 Hz) inversely modulated Go and No-Go N1, but had no impact on Go or No-Go P3 [16]. The most robust of these findings has been the direct link between prestimulus alpha amplitude and the P3 observed in numerous studies [13,14,16].

### 1.2. Related Work

Following our successful use of t-PCA in decomposing ERPs, we turned to the use of frequency principal component analysis (f-PCA) to move beyond the use of band-limited EEG [17]. Our first brain dynamics study using f-PCA for prestimulus EEG component separation and t-PCA for ERP decomposition [18] broadly confirmed the differential impacts of prestimulus alpha and beta amplitudes on Go N1 and P3b, and No-Go N1 and P3a, but with clarifying enhancements provided by the separation of different components in each of the alpha and beta bands. We recently extended this approach by publishing a novel developmental brain dynamics study in which we examined prestimulus EEG/ERP/behaviour linkages in groups of children, young adults, and older adults [19].

**Figure 1:**
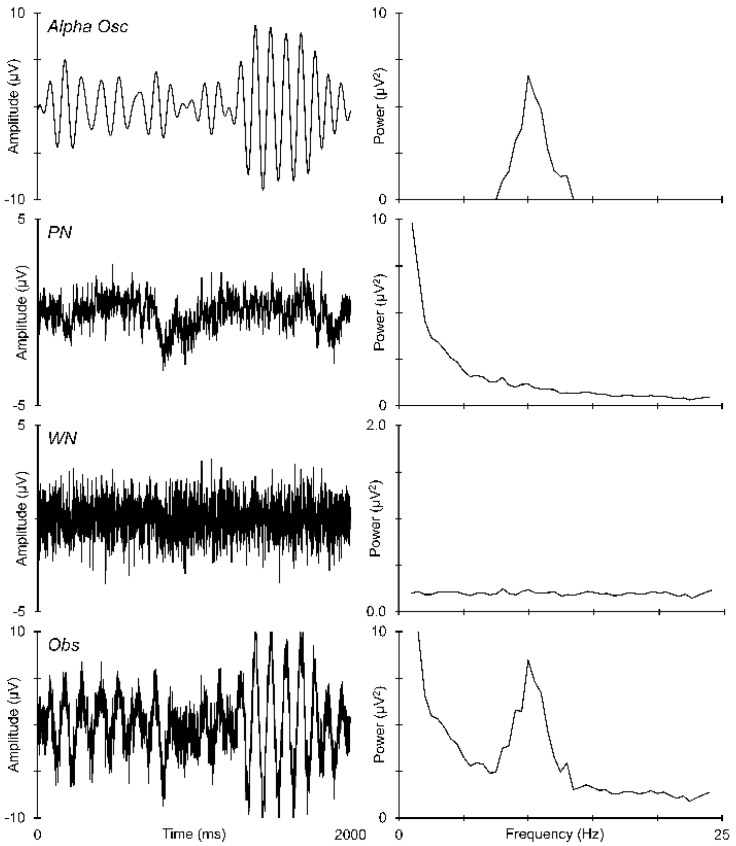
Different ways to view EEG activity, with voltage as a function of time on the left, and power as a function of frequency on the right. The **left** panel shows simulated 2 s epochs of data, while the **right** panel shows the frequency spectrum of that epoch. Illustrative data are shown for alpha oscillations, pink noise (PN), white noise (WN), and their observed sum (Obs).

However, the EEG frequency spectrum contains not only oscillations but non-oscillations—voltage fluctuations that are not rhythmical or repetitive. Although these are commonly considered as nuisance noise, some better-defined non-oscillations have been increasingly considered for their possible functional roles in brain dynamics. Chief amongst these non-oscillations was described by Johnson [20] in his studies of vacuum-tube Schottky noise. This non-random noise, with power proportional to the inverse of the frequency (i.e., 1/*f*), is now termed “pink” noise (PN). Another well-defined non-oscillation found in the EEG is “white” noise (WN), which has a uniform power distribution over frequency [21]. Figure 1 illustrates how PN and WN can add to alpha oscillations in the time domain (left panel) and contribute to the observed frequency spectrum (right panel).

**Figure 2:**
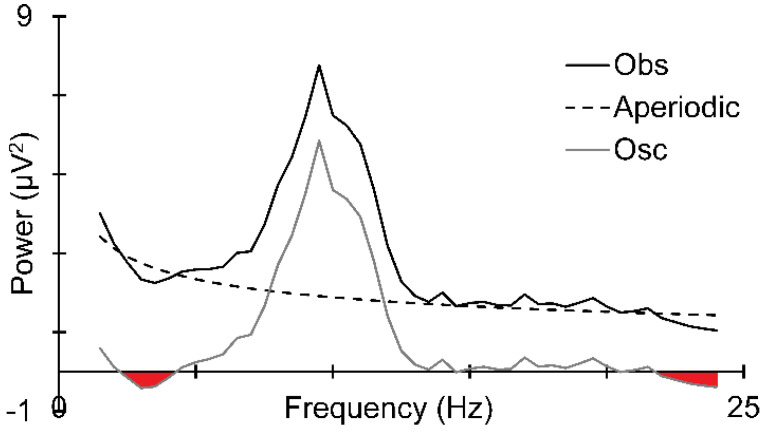
An epoch from observed EEG activity (Obs) decomposed using *specparam*. The aperiodic noise obtained (Aperiodic) is greater than the observed input data in some frequencies, a physical impossibility leading to negative power estimates (coloured red) at some frequencies in the oscillations (Osc) derived by subtraction (Obs—Aperiodic).

Efforts have been made to estimate PN in the EEG, but these have not been without issues. For example, the most widely used extraction method, *specparam* [22], extracts a *near-pink* noise (i.e., the extracted noise power only approximates a 1/*f* distribution) that is now labelled “aperiodic” noise. This often contains more power at some frequencies than the observed spectrum [21]. Consequently, if this is subtracted from the observed power, there is an under-estimate of the oscillation power, producing instances of *negative power* (which is physically impossible). These problems are illustrated in Figure 2, in which a genuine observed EEG spectrum (Obs) from a participant in the present study was decomposed using *specparam* to obtain the aperiodic noise. The negative power estimates in the derived oscillation (Osc) are indicated in red.

Barry and De Blasio [21] published an algorithm (pink and white noise extractor: *PaWNextra*) that can provide valid estimates of both PN and WN in EEG spectra. The potential role of such valid noise estimates in perceptual and cognitive processing has not yet been explored. We use that algorithm here to estimate PN and WN in the immediately-prestimulus EEG of participants in an equiprobable auditory Go/No-Go task. After subtracting these valid estimates of noise, we decompose the immediately-prestimulus EEG oscillation spectra using f-PCA [17,18]. We then use these EEG oscillation components and PN and WN estimates to explore how they predict the behavioural measures and ERP components in the equiprobable Go/No-Go task. The ERP components are derived using separate Go and No-Go t-PCAs to avoid misallocation of variance [23]. As far as we know, this approach, combining frequency and temporal PCAs with valid PN and WN estimates, has not been used before in a study of brain dynamics.

### 1.3. Key Contributions of This Study

- Examines within-task prestimulus EEG effects on ERPs and behaviour in an auditory equiprobable Go/No-Go task.
- Validly estimates and removes pink and white noise from the observed EEG, leaving the rhythmical oscillations.
- Extracts the noise-free EEG oscillation components using frequency PCA.
- Decomposes the Go and No-Go ERP components by separate temporal PCAs, avoiding misallocation of variance.
- Uses regression to search for novel within-task EEG/ERP/behaviour links.

## 2. Materials and Methods

### 2.1. Participants

Undergraduate students from the School of Psychology, University of Wollongong, volunteered to participate in exchange for course credit. All gave written informed consent in line with a protocol approved by the joint University of Wollongong/South East Sydney and Illawarra Area Health Service Human Research Ethics Committee (Ethics Number 2009/220 Initial approval July 2009, last renewal August 2024). Participants were screened for handedness, hearing level, abstinence from caffeine and tobacco for a minimum of 2.5 h prior to their testing session, and their health status. Participants who reported prior head trauma resulting in unconsciousness, epileptic seizures, prior or current psychiatric illness, and/or psychoactive drug use were excluded. We began with 50 participants but lost 3 due to poor quality EEG/ERPs, leaving 30 females and 17 males aged 18–31 years (*M* = 20.9, *SD* = 3.2); all self-identified as right-handed.

### 2.2. Task and Procedure

Following informed consent and screening, participants were fitted with electrophysiological recording equipment and sat in a quiet and dimly lit testing room. Continuous EEG was recorded while participants completed a visual EOG calibration task (following Croft and Barry, 2000 [24]), and two blocks of an equiprobable auditory Go/No-Go task. The auditory stimuli were 1000 Hz and 1500 Hz tones, each of 80 ms duration (including 15 ms rise and fall times) and played over circumaural headphones (Sony MDR-V700) at 60 dB SPL, presented at a fixed stimulus onset asynchrony of 1100 ms (i.e., each stimulus started 1100 ms after the start of the previous stimulus). Each block contained 75 Go and 75 No-Go tones that were shuffled to obtain a random presentation order that varied between participants. The tone assigned as the Go stimulus alternated between the blocks. Participants responded to the Go stimulus with a button-press using their dominant hand. Brief breaks were offered between blocks to reduce fatigue; these were untimed but usually lasted one or two minutes.

### 2.3. Electrophysiological Recording

EEG data were recorded from 30 scalp sites (Fp1, Fp2, F7, F3, Fz, F4, F8, FT7, FC3, FCz, FC4, FT8, T7, C3, Cz, C4, T8, TP7, CP3, CPz, CP4, TP8, P7, P3, Pz, P4, P8, O1, Oz, O2), and EOG data were recorded from four facial electrodes positioned above/below the left eye, to capture vertical activity, and beyond the outer canthus of each eye, to capture horizontal activity. Data were recorded (DC to 70 Hz) at 1000 Hz using a Neuroscan Synamps 2 amplifier with Neuroscan Acquire software (Compumedics Limited, Version 4.3.1) with M1 serving as the online reference, and M2 recorded as a separate channel.

### 2.4. EEG Quantification

EEG data were EOG corrected using the RAAA correction procedure [24] as implemented in MATLAB (The MathWorks, Inc., R2019b) using scripts provided by Tony [25]. Using EEGLAB [26] v2022.0, the EOG-corrected data were re-referenced to digitally linked mastoids and bandpass filtered 0.1 to 30 Hz (non-causal, zero-phase shift). The data were then visually inspected to identify channels that dropped out or flatlined, and these were replaced using spherical spline interpolation. For valid trials, epochs −500 to 500 ms around each stimulus onset were extracted, and a three-step automatic artefact rejection procedure was run similar to Foti et al. [27]; this process rejected epochs detected as having extreme amplitudes > 125 µV, voltage jumps > 50 µV between datapoints, and those with a maximum voltage change < 0.05 µV in any 100 ms period. Go and No-Go trials with a response in their 500 ms prestimulus period were identified as invalid trials and were discarded. Valid Go trials were Go stimuli with a button-press response within the 100 to 500 ms poststimulus period (i.e., not contaminating the subsequent prestimulus period with a late button press). Valid No-Go trials had a No-Go stimulus with no button press in the subsequent interstimulus period. Participants were required to have at least 50 valid Go and 50 valid No-Go trials for inclusion. To maintain the signal-to-noise ratio within subjects between conditions, the number of accepted epochs was matched between Go and No-Go. This number was set (within subject) by the condition with the lower number of accepted trials, and the corresponding number of trials was then randomly selected for analysis in the alternate condition.

Prestimulus EEG epochs (−500 to 0 ms) at each electrode were zeroed across the epoch to remove DC offsets, windowed with a 10% Hanning window, padded to 1000 data points, and submitted for discrete Fourier analysis to obtain EEG spectra with 1 Hz resolution. EEG magnitudes (μV) were corrected for the zero-padding and Hanning window, and the mean (within subject) spectra were derived for each condition and site. As the Go and No-Go stimulus presentation was completely random and their ERPs were not substantial at the beginning of this epoch (600 ms after the preceding stimulus), the Go and No-Go EEG prestimulus spectra were not expected to differ. Hence, the mean Go/No-Go spectra from 2 to 24 Hz for each participant were submitted to *PaWNextra* [21] (available at https://forms.gle/XEm1VdoBE4MnYhUS8), and the PN and WN estimates were obtained. The *PaWNextra* solutions were used to generate the PN and WN spectra across the broader 1 to 30 Hz frequency range. These were subtracted from the mean Go/No-Go EEG spectra (within subjects) to obtain the noise-corrected prestimulus oscillation spectra for each participant, which were submitted to f-PCA using the ERP PCA Toolkit [28] with the covariance matrix as input. There were 1410 cases (47 participants × 30 electrodes) and 31 variables (1 Hz steps from 0 to 30 Hz), giving a case/variables ratio of 45.5. Following Barry, De Blasio, and Karamacoska [17], all 31 components were extracted and rotated using Promax. Similar to prior studies [17,18,21,23], substantial frequency components were selected for analysis using a minimum variance cut-off. Here we used ≥1.5% variance, following [19]; subsequently the within-subject global mean amplitude was extracted for each component.

### 2.5. ERP Quantification

The ERP period (−100 to 500 ms) was extracted from the valid EOG-corrected artifact-free epochs, and the mean Go and No-Go ERPs were derived within subjects. The average ERPs were half-sampled to 500 Hz and submitted to separate t-PCAs for each condition (Go, No-Go). For each t-PCA, the input comprised 1410 cases (47 participants × 30 channels × 1 condition) and 300 variables (−100 to 500 ms at 2 ms resolution), providing a cases-to-variables ratio of 4.7. The t-PCAs were conducted with the covariance matrix, Kaiser normalisation, and unrestricted Varimax rotation of 300 components following recommendations in Barry, De Blasio, Fogarty, and Karamacoska [23]. Temporal components accounting for ≥1.5% variance were considered for analysis, and the within-subject global mean peak amplitude was extracted separately for each Go and No-Go component identified.

### 2.6. Statistical Analyses

To explore the EEG component determinants of the Go and No-Go behavioural measures and ERP components, we conducted separate stepwise multiple regressions. These used the prestimulus oscillation component amplitudes, plus PN and WN amplitudes at 1 Hz, as independent variables, and the behavioural and ERP amplitudes as dependent variables. For each EEG/ERP and PN/WN variable, the global mean amplitude across all sites was used. This mean amplitude measure uses information from all sites across the scalp, and avoids distortions associated with selection of data from within an arbitrary region of interest.

## 3. Results

### 3.1. Accepted Trials

Accepted participants had 50 to 148 trials (*M* = 112.6, *SD* = 22.3) for analysis in each of the Go and No-Go conditions.

### 3.2. Behaviour

In response to the Go stimuli, there were 0 to 20% omission errors (*M* = 4.2%, *SD* = 5.1%), with an additional 1.3 to 52.0% slow RT errors (RT > 500 ms; *M* = 14.4%, *SD* = 9.5%), and 0 to 4.7% fast RT errors (RT < 100 ms; *M* = 0.7%, *SD* = 1.1%). The mean RT was 339.8 ms (*SD* = 27.6), and RT variability (i.e., within-subject RT *SD*) was 70.2 ms (*SD* = 10.3). For No-Go stimuli, there were 0 to 28.9% (*M* = 6.2%, *SD* = 6.2%) commission errors.

### 3.3. Prestimulus EEG

**Figure 3:**
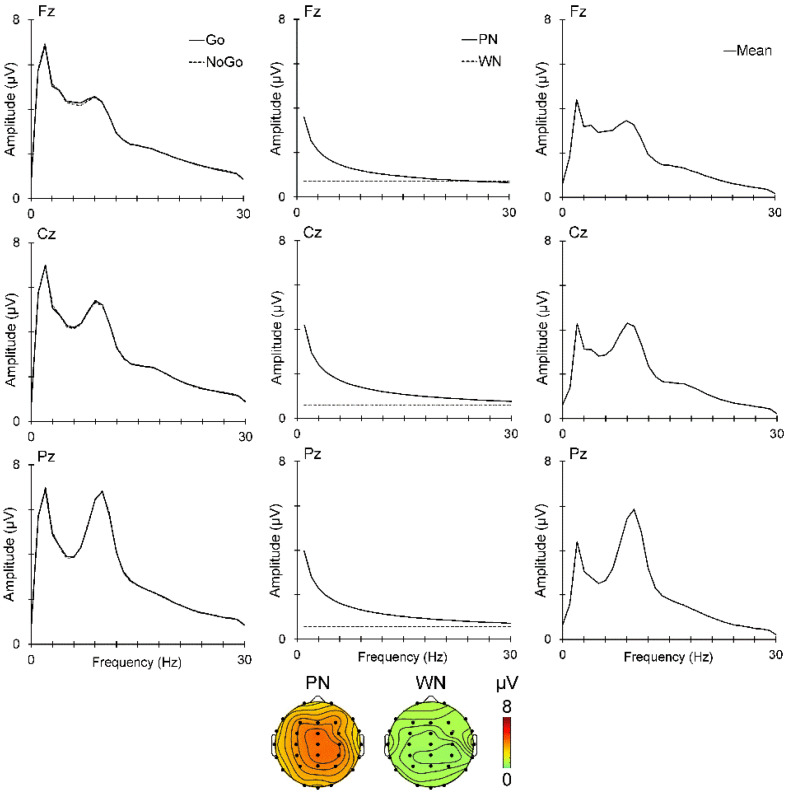
**Left**: Mean prestimulus amplitude spectra at the midline sites for Go and No-Go. There is almost complete overlap. **Centre**: Mean amplitude spectra for PN and WN. **Right**: Mean amplitude spectra for the noise-free oscillations. **Bottom**: Topographic head maps for noise amplitudes at 1 Hz.

The EEG amplitude spectra at the midline sites are shown in Figure 3, with the raw data on the left, PN and WN in the centre, and noise-free oscillations on the right. The raw spectra for both Go and No-Go are overlaid, but are barely distinguishable, confirming our expectations and supporting their averaging for input into *PaWNextra* and subsequent processing.

**Figure 4:**
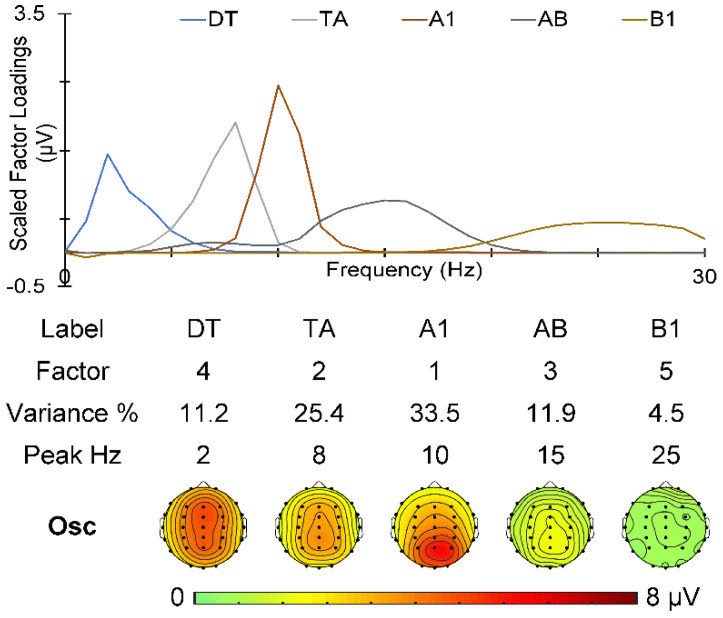
Frequency PCA decomposition of the noise-free mean Go/No-Go prestimulus oscillations. The scaled factor loadings as a function of frequency are shown above details of the components in frequency order.

The spectra for PN and WN appear as expected, and their topographies at 1 Hz are shown at the bottom of Figure 3. PN shows a vertex distribution, while WN is reduced in magnitude and more diffuse, comparable with [21]. The Go/No-Go prestimulus spectrum after removal of PN and WN was submitted to f-PCA with Promax rotation and yielded five oscillation components carrying more than 1.5% of the variance each, for a total of 86.5% variance. The scaled factor loadings and their topographic distributions are shown in Figure 4. There was one delta/theta (DT) component peaking at 2 Hz, one theta/alpha (TA) component peaking at 8 Hz, an alpha (A1) component with a 10 Hz peak, an alpha/beta (AB) component peaking at 15 Hz, and a beta component (B1) peaking at 25 Hz. The topographies shown are similar to those previously published [15,16,17,18,19,20] and support the present band labels.

### 3.4. Poststimulus ERPs

**Figure 5:**
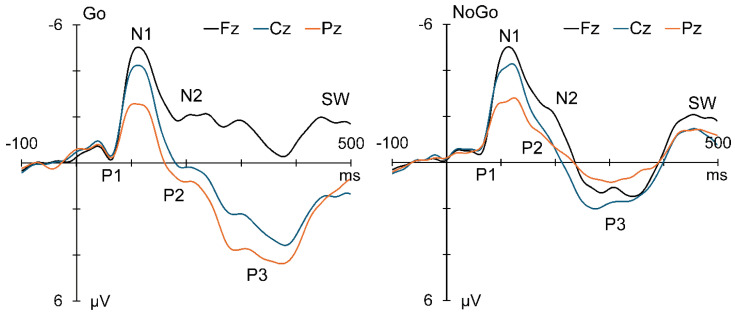
Morphology of the mean ERPs at the midline sites. **Left**: Go ERPs; note large parietal P3. **Right**: Corresponding No-Go ERPs; note reduced P3 dominant at Cz.

The mean ERPs at the midline sites are displayed in Figure 5, with Go ERPs on the left and No-Go on the right. The main components are labelled on the plots.

**Figure 6:**
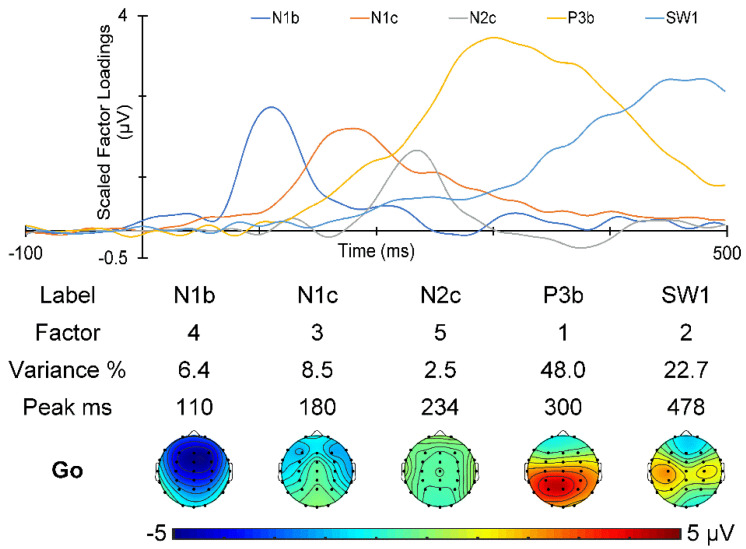
Temporal PCA decomposition of the Go ERPs. The scaled factor loadings as a function of time are shown above details of the Go components in temporal order.

The t-PCA of the Go ERPs identified five components carrying more than 1.5% of the variance each, for a total of 88.1% variance. Their scaled factor loadings and topographies are shown in Figure 6. Go components N1b, N1c, N2c, P3b, and SW1, in temporal order, were identified by their characteristic latency and topography [2,4,7,29,30].

**Figure 7:**
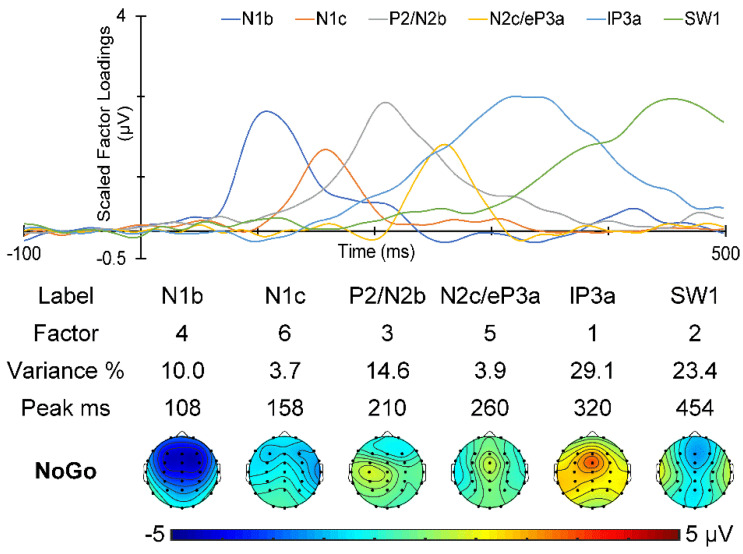
t-PCA decomposition of the No-Go ERPs. The scaled factor loadings are shown as a function of time above details of the No-Go components in temporal order.

The No-Go t-PCA identified six components carrying more than 1.5% of the variance each, for a total of 84.8% variance. Their scaled factor loadings and topographies are shown in Figure 7. Their characteristic latency and topography [2,4,7,29,30] identified No-Go components in temporal order: N1b, N1c, a composite P2/N2b, a composite N2c/early P3a (*e*P3a), a late P3a (*l*P3a), and an SW1.

### 3.5. Regression Outcomes

**Figure 8:**
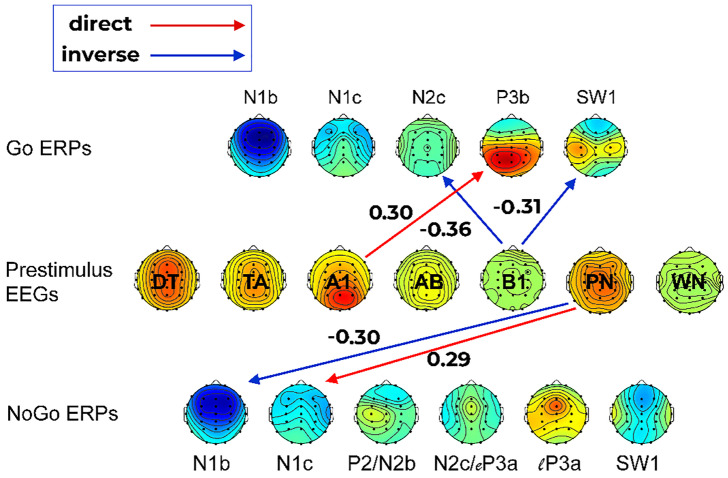
Significant regression outcomes showing direct and inverse links between the mean Go/No-Go prestimulus EEG oscillation components and pink noise (PN) and white noise (WN) measures, and subsequent Go and No-Go ERP components.

EEG oscillation and noise predictors (DT, TA, A1, AB, B1, PN, WN) were not associated with any behavioural measures (Go omission errors, slow/fast RT errors, RT [mean or variability], or No-Go commission errors). However, the prestimulus EEG predictors were associated with some Go and No-Go ERP components, as shown in Figure 8. Go N2c amplitude was inversely dependent on B1 (*β* = −0.36, *t* = −2.57, *p* = 0.013); that is, as B1 increased, the negativity in N2c also increased. Go P3b was directly dependent on A1 amplitude (*β* = 0.30, *t* = 2.11, *p* = 0.040). The positive SW1 was inversely dependent on B1 amplitude (*β* = −0.31, *t* = −2.22, *p* = 0.032); that is, as B1 amplitude increased, Go SW1 positivity became smaller. The early No-Go N1b was inversely dependent on PN amplitude (*β* = −0.30, *t* = −2.14, *p* = 0.038); i.e., increasing PN amplitude resulted in larger (more negative) No-Go N1b amplitudes. By contrast, No-Go N1c was directly dependent on PN (*β* = 0.29, *t* = 2.03, *p* = 0.048), indicating that, as PN increased, the magnitude of No-Go N1c became smaller (i.e., less negative).

As a check for any secondary links, the behavioural measures were regressed on the EEG-affected ERP measures. Go N2c inversely predicted mean RT (*β* = −0.32, *t* = −2.29, *p* = 0.027); larger (more negative) N2c was associated with greater RT. Go P3b inversely predicted slow RT errors (*β* = −0.36, *t* = −2.56, *p* = 0.014) and RT variability (*β* = −0.41, *t* = −3.04, *p* = 0.004); i.e., greater P3b was linked to fewer slow RT errors and reduced RT variability. The PN-affected No-Go N1 components were not linked to No-Go commission errors.

## 4. Discussion

The major brain dynamics thrust of this study was to link the immediately-prestimulus EEG to the following ERP components and behaviour. Essentially this links the brain’s stimulus-elicited response to the EEG activity during the 500 ms epoch prior to stimulus onset. This within-task approach to estimating the EEG/ERP link was recently pioneered in a developmental brain dynamics study [19]. We further extended this approach here by examining the use of a relatively new method to remove valid PN and WN (*PaWNextra* [21]) from the immediately-prestimulus EEG activity during an equiprobable auditory Go/No-Go task. The aim was to explore how the noise-free prestimulus oscillations, as well as PN and WN, affect stimulus-evoked behaviour and ERPs. This is the first formal application of *PaWNextra* since its publication in 2021, and this novel foundation is built upon by our use of the optimised f-PCA to extract data-driven components from the noise-free EEG oscillation spectra [17], and our use of separate t-PCAs to extract the Go and No-Go ERP components [23].

### 4.1. EEG Predictors

We randomly presented the equiprobable Go and No-Go stimuli by shuffling the stimulus order in each stimulus block. This is important as it allows the assumption that there will be no difference in the prestimulus activity immediately before the Go vs. No-Go stimulus. The near-identical overlaid prestimulus spectra for the Go and No-Go stimuli, shown on the left in Figure 3, supported that expectation. Their mean spectra were used to estimate PN and WN (middle panel of Figure 3) and their subtraction yielded the mean prestimulus oscillation spectra (Figure 3 right panel). In turn, this allowed for the use of a single f-PCA to decompose the noise-free oscillation spectra (see Figure 4), our primary EEG focus here.

The noise-free prestimulus EEG yielded five components: a complex delta/theta component (DT), a theta/alpha component (TA), one alpha component (A1), a complex alpha/beta (AB) component, and one beta component (B1). These cannot be compared with published EEG components as there are few immediately-prestimulus in-task examples published to date, and no prior noise-free examples. Also, the PN and WN obtained here are the first within-task valid noise estimates derived from the immediately-prestimulus observed EEG. Our previous noise head maps were obtained under resting (non-task) conditions [21]. Despite this, their topographies are somewhat similar, with PN showing a vertex distribution, and WN showing a frontal/parietal distribution. These seven measures (five noise-free oscillation components, PN, and WN) comprised our EEG predictors.

### 4.2. Dependent Variables

We examined six behavioural dependent variables: Go omission errors, slow/fast RT errors, the mean and variability of Go RT, and the rate of No-Go commission errors.

The Go ERPs generated five components: N1b, N1c, N2c, P3b, and SW1, while the No-Go ERPs contained six familiar components: N1b, N1c, P2/N2b, N2c/early P3a, late P3a, and SW1. These corresponded well with our general expectations (e.g., [4,23,29] and the young adult group in [19]).

### 4.3. Regression Analyses

In this exploratory study, we carried out a series of stepwise multiple regressions, using our seven EEG measures as predictors for our seventeen (behavioural and ERP) dependent variables. Only five regressions indicated significant links between the independent and dependent variables.

Of the five Go ERP components, only those associated with the imperative Go response were linked to prestimulus EEG oscillations, clarifying and expanding on our prior findings [18]. The P3b component and its precursor N2c, long-established markers of active Go responding, were respectively enhanced by greater alpha amplitude and beta amplitude in the prestimulus oscillations. The subsequent SW positivity was reduced by greater beta amplitude. This suggests that the greater fast wave oscillations in the prestimulus period facilitated the production of the Go response, with its ERP markers, and reduced the need for post-performance evaluation reflected in the SW component. That is, greater alpha and beta activity at stimulus onset resulted in more efficient Go responding. Neither PN nor WN impacted the Go processing chain.

Unlike our earlier investigation [18], the six No-Go ERP components were not linked to any prestimulus EEG oscillations at stimulus onset, suggesting that our prior findings may be attributable to the undifferentiated inclusion/amalgamation of pink noise and white noise in the prestimulus frequency components. However, the early N1b and N1c components observed here, associated with the sensory processing of the stimulus leading to its evaluation as a No-Go stimulus, were each impacted by PN. Greater PN amplitudes were linked to larger No-Go N1b and smaller No-Go N1c. The N1b component has been suggested as the primary marker of tone frequency discrimination [30,31], so it appears that larger PN contributes to successful and/or more efficient No-Go discrimination in this task. The N1c component has been linked to stimulus–response processing in paradigms that require a response [30], so its reduction by PN here in the No-Go processing stream suggests a further PN contribution to No-Go processing. We have previously speculated that similar functional enhancements in sensory processing outcomes may reflect proactive (cf. reactive) processes (see the Supplementary Materials in [32]), thus is it plausible that prestimulus PN may directly reflect this top–down control process. Similar interpretations have been reported in other contexts using alternate (suboptimal) approaches to 1/*f* measurement [33]. Although not possible here, future research could explore the feasibility of this speculation by introducing a bias in Go/No-Go probabilities to test whether the PN influence on ERP components scales with cognitive control demands.

None of the behavioural measures were directly linked to the prestimulus EEG oscillation components or noise measures. However, the EEG-affected Go N2c and P3b ERPs were related to Go performance measures. Larger N2c was associated with greater RT, and greater P3b was linked to fewer slow RT errors and reduced RT variability. These interesting indirect EEG/ERP/behaviour effects of prestimulus oscillations need further investigation in future studies.

Significant changes from our previous results [19] indicate the importance of considering the impact of non-oscillations in the EEG measures. At least some of the previously noted EEG/ERP/behaviour linkages must reflect non-oscillations. The novelty of the previous and current studies demands further replication to clarify the situation.

### 4.4. Limitations

This exploratory study carried out a number of regressions seeking predictors of the behavioural responses and the Go and No-Go ERP components. As all the predictor variables were completely novel, we were not concerned about issues of multiple testing in this exploratory investigation [34]. Our results provide outcomes that can be used in future studies, where testing of a narrower range of predictions based on the present results will require alpha adjustments to account for the effects of multiple testing.

In addition, it is possible that some of the negative findings here could be a reflection of insufficient power. We were not able to carry out a proper *a priori* power analysis in the absence of the expected effect sizes for the novel linkages tested. We began our study with a fairly large group for a brain dynamics study (*N* = 50), but loss of participants with poor EEG data reduced this to 47. We considered this more than adequate, as our previous dynamics study [19] used an a priori determined group size of 20. The present results will allow informed power calculations for future work linking noise-free oscillations and noise amplitudes to outcomes in the Go/No-Go task.

### 4.5. Beyond the Auditory Go/No-Go Task

The novel package of techniques used here is applicable in many ERP studies where the interplay between the ongoing EEG oscillations and stimulus processing is of interest. Here, we used a fixed stimulus onset asynchrony (1100 ms) of sufficient duration to allow the stimulus-induced ERP to return to baseline EEG levels before presenting the next stimulus event, and we checked this by comparing the prestimulus EEG spectra from before our two stimuli (Go and No-Go). Apart from that minor timing restriction, this approach can be followed in a wide range of paradigms used in cognitive and affective neuroscience.

### 4.6. Conclusions

The present study suggests that alpha and beta oscillations are important in generating the imperative Go ERP components marking elicitation, execution, and evaluation of the Go response in the equiprobable auditory Go/No-Go task. By contrast, these had no discernible links to No-Go processing. Rather, pink noise immediately before stimulus onset, perhaps reflecting proactive control processes, enhanced the early N1b component, the major marker of tone discrimination, facilitating identification of the stimulus as No-Go. The subsequent N1c, important in relation to Go processing, was decreased by the same pink noise enhancement, perhaps reflecting a saving of cortical resources not needed in the No-Go processing stream. These findings need replication in further studies, but their observation in the present study points to the value of the novel data processing approach employed here, and its promising use to elucidate the oscillatory correlates of sensory and cognitive control processes.

Currently, we know very little of the origins or functionality of pink noise in the human EEG. The present results, indicating a significant role for pink noise in early No-Go stimulus processing, strongly suggest the need for further basic research into pink noise. We reiterate that this is the first application of a valid pink noise estimator in a task related study of scalp EEG. Such a valid estimation is a necessary precursor for the detailed study of pink noise, its generation in the brain, and its effects in relation to human cognition and perception.

## Data Availability

The data presented in this study are available on request from the corresponding author.
